# Interconnected sub-networks of the macaque monkey gustatory connectome

**DOI:** 10.3389/fnins.2022.818800

**Published:** 2023-02-16

**Authors:** Renée Hartig, Ali Karimi, Henry C. Evrard

**Affiliations:** ^1^Max Planck Institute for Biological Cybernetics, Tübingen, Germany; ^2^Functional and Comparative Neuroanatomy Laboratory, Werner Reichardt Centre for Integrative Neuroscience, Eberhard Karl University of Tübingen, Tübingen, Germany; ^3^Department of Psychiatry and Psychotherapy, University Medical Center, Johannes Gutenberg University Mainz, Mainz, Germany; ^4^Center for Biomedical Imaging and Neuromodulation, Nathan Kline Institute for Psychiatric Research, Orangeburg, NY, United States; ^5^Department of Connectomics, Max Planck Institute for Brain Research, Frankfurt, Germany; ^6^International Center for Primate Brain Research, Center for Excellence in Brain Science and Intelligence Technology, Institute of Neuroscience, Chinese Academy of Sciences, Shanghai, China

**Keywords:** functional connectivity, neuroimaging, insular cortex, interoception, taste, non-human primate

## Abstract

Macroscopic taste processing connectivity was investigated using functional magnetic resonance imaging during the presentation of sour, salty, and sweet tastants in anesthetized macaque monkeys. This examination of taste processing affords the opportunity to study the interactions between sensory regions, central integrators, and effector areas. Here, 58 brain regions associated with gustatory processing in primates were aggregated, collectively forming the *gustatory connectome*. Regional regression coefficients (or β-series) obtained during taste stimulation were correlated to infer functional connectivity. This connectivity was then evaluated by assessing its laterality, modularity and centrality. Our results indicate significant correlations between same region pairs across hemispheres in a bilaterally interconnected scheme for taste processing throughout the gustatory connectome. Using unbiased community detection, three bilateral sub-networks were detected within the graph of the connectome. This analysis revealed clustering of 16 medial cortical structures, 24 lateral structures, and 18 subcortical structures. Across the three sub-networks, a similar pattern was observed in the differential processing of taste qualities. In all cases, the amplitude of the response was greatest for sweet, but the network connectivity was strongest for sour and salty tastants. The importance of each region in taste processing was computed using node centrality measures within the connectome graph, showing centrality to be correlated across hemispheres and, to a smaller extent, region volume. Connectome hubs exhibited varying degrees of centrality with a prominent leftward increase in insular cortex centrality. Taken together, these criteria illustrate quantifiable characteristics of the macaque monkey gustatory connectome and its organization as a tri-modular network, which may reflect the general medial-lateral-subcortical organization of salience and interoception processing networks.

## Introduction

Neural circuits in the brain process sensory signals generated across multiple modalities, with such processing occurring at various relay stations along distinct as well as converging afferent pathways (Van Essen et al., 1992). In these circuits, the subcortex gates, relays, and coordinates interactions between cortical regions and downstream effector targets (McCormick and Bal, 1994; Thorn and Graybiel, 2010; Whitmire et al., 2016), and the cerebral cortex integrates multi-modal sensory information alongside information history updating and ongoing neural activity, lending itself to the dynamicity of “brain states” (Rao and Ballard, 1999; Hesselmann et al., 2008; McCormick et al., 2015; Morcos and Harvey, 2016). Examining the interactions across subcortical and cortical regions within the context of taste processing provides a basis to survey the gustatory connectome and its relation to bottom-up and top-down processing streams.

Human neuroimaging studies of functional connectivity have revealed a dynamic interplay between regions (Cottam et al., 2018; Fransson and Thompson, 2020; Fukushima and Sporns, 2020) that results in network activity modulation across different brain states (e.g., sensory processing, default mode or executive function). To help further understand the gustatory connectome, we examined network hubs, generally characterized by their central placement in the network and high degree of connectivity to other regions (or nodes in the context of graph theory) (van den Heuvel and Sporns, 2013). The nodes of the gustatory connectome coalesce around prior anatomical and functional work on taste processing in primates. Electrophysiological studies in macaque monkeys have uncovered taste-responsive neuronal populations in the brainstem (solitary tract nucleus, NTS) (Yaxley et al., 1985; Scott et al., 1986b), in the thalamus (basal part of the ventromedial nucleus, VMb) (Pritchard et al., 1986), and in the granular dorsal fundus of the insular cortex, which is the cortical terminus of medullo-thalamo-cortical gustatory afferents (Beckstead et al., 1980; Yaxley et al., 1990; Plata-Salamán and Scott, 1992; Scott and Plata-Salamán, 1999; Scott et al., 1999). Human functional magnetic resonance imaging (fMRI) and positron emission tomography (PET) studies have further corroborated the role of the insular cortex and adjacent opercula in taste processing (Faurion et al., 1998; Small et al., 1999; Ogawa et al., 2005; Spetter et al., 2010). These studies demonstrated evidence for a primary sensory-based representation of taste afferents in the middle dorsal fundus region of the human insula (Avery et al., 2020) which, we proposed, is homologous with the simian middle dorsal fundus (for a review see Evrard, 2019).

In addition to the primary sensory representations, the central integration of taste involves poly-modal cortical regions, such as the orbitofrontal cortex (Rolls and Baylis, 1994), as well as higher-order integrators that form an aggregate network containing sub-regions of the anterior cingulate (Bush et al., 2000), anterior insular (Lamm and Singer, 2010; Medford and Critchley, 2010; Gu et al., 2013; Wang et al., 2019; Wu et al., 2019), frontal (Enel et al., 2020), and posterior medial cortices, including the precuneus (Cavanna and Trimble, 2006). Furthermore, as sensory processing elicits a cascade of regional recruitment, several cortical and subcortical regions associated with affective and emotional aspects of sensory processing are also involved (Allen et al., 1991; Carmichael and Price, 1995; Price, 2007). To complete the loop of bottom-up and top-down processing, effector targets (e.g., parabrachial complex and substantia nigra, Reilly et al., 1993; Pritchard et al., 2000; Grillner and Robertson, 2015) are also included. Nevertheless, how all these regions functionally connect with one another in the context of taste processing remains unclear.

The present study probed the organization of the taste processing connectome in the anesthetized macaque monkey by mapping beta (β) series correlations (Göttlich et al., 2015) derived from event-related fMRI data using sour, salty, and sweet taste stimuli. This seed-free, event-related examination of brain connectomics, applied previously both in humans (Rissman et al., 2004) and rodents (Winkelmeier et al., 2022), interprets region pairs whose β-series are correlated to be functionally connected, as their attributable blood-oxygen-level-dependent (BOLD) response to the sensory input follows a similar pattern.

After an initial mapping, the macaque monkey gustatory (taste) connectome was evaluated by assessing its laterality, modularity and centrality. More particularly, we aimed (1) to uncover interactions underlying the processing of taste between the two hemispheres of the brain (laterality); (2) to determine whether the connectome partitioned into subsets of regions strongly connected together and, if so, how these sub-networks may be impacted by taste stimuli (modularity); and finally, (3) to identify connectome hubs that might play a role in regional interactions in the brain (centrality). Taken together, this work further elucidates the relationship between connectome nodes and how taste quality-specific processing may be reflected across modular sub-networks, perhaps shared across all salient information processing.

## Results

### General observations

**FIGURE 1:**
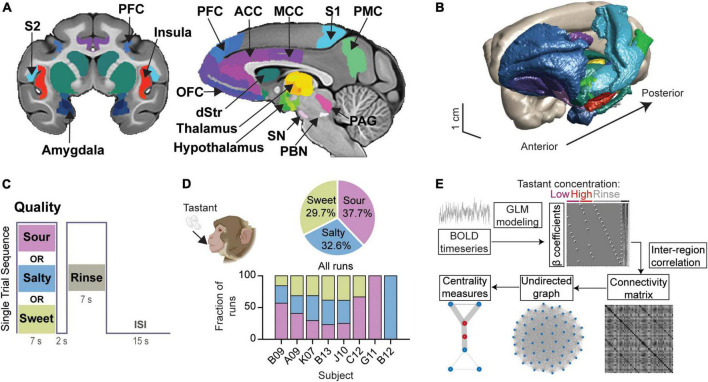
Modeling the gustatory connectome in the adult macaque monkey. **(A)** Coronal and mid-sagittal sections from the base template image of the CHARM and SARM macaque monkey brain atlases indicating the regions included in the gustatory connectome. ACC, anterior cingulate cortex; dStr, dorsal striatum (caudate and putamen); MCC, mid-cingulate cortex; OFC, orbitofrontal cortex; PAG, periaqueductal gray; PBC, parabrachial complex; PFC, prefrontal cortex; PMC, posterior medial cortex (area 7m, precuneus); S1, primary somatosensory cortex; S2, secondary somatosensory cortex; SN, substantia nigra. **(B)** A 3D surface view of the macaque monkey taste connectome, with regions colored as shown in panel **(A)**. **(C)** Stimulus block design for a single trial sequence. Each run consisted of 15 tastant (pseudorandomized low and high concentration presentation) and 15 rinse solution delivery blocks. An inter-stimulus interval (ISI) of 15 sec separates each trial of taste-rinse presentation. **(D)** Pie chart of the percentage of runs per taste quality (upper panel, *n* = 104, 90, 82 for sour, salty, and sweet, respectively). The number of runs for each subject were: B09, *n* = 51; A09, *n* = 54; K07, *n* = 51; B13, *n* = 52; J10, *n* = 44; C12, *n* = 12; G11, *n* = 7; B12, *n* = 5. The fraction of runs for each taste quality across animals is illustrated by the bar plot (lower panel). Animal cartoon (upper left) adapted from BioRender.com. **(E)** Schematic demonstrating the steps involved in deriving the β coefficients, connectivity matrix and graph centrality measures for the gustatory connectome. Representative design matrix used for estimating β coefficients (upper). The undirected connectivity matrix (lower middle) was generated from the BOLD signal through general linear modeling and β-series correlation. Runs were modeled analogously for three event types (low-, high-concentration tastant, and rinse solution) across taste qualities (sour, salty, and sweet).

We studied the functional connectome of taste processing in the anesthetized rhesus macaque monkey (*n* = 8). This gustatory connectome coalesces around 29 bilateral regions (58 regions in total) (Figures 1A,B and Supplementary Table 2) selected mainly based upon prior research implicating them in taste processing. Of the 29 bilateral regions, five regions were included either to fill spatial gaps between neighboring connectome regions (i.e., para-insular area, *paraIns*; precentral opercular area, *PrCo*) or to explore further their individual role in the taste network (i.e., parietal area 3b; posterior orbitofrontal cortex area 13, *pOFC*; and retro-insula, *Ri*). Gustatory connectome regions were rendered in the three-dimensional space of the macaque monkey structural template NMT v2 (Figure 1B). The center-of-mass coordinates for each of the regions are provided in Supplementary Table 2.

The functional connectivity within the gustatory connectome was studied by applying sour, salty, or sweet taste stimuli to the tongue during fMRI. The different tastants were presented in separate fMRI runs (or scans), with each run devoted to one taste quality only. A single run was composed of 15 trials, with each trial consisting of a 7 sec delivery of tastant, followed by a 2 sec pause, and a 7 sec rinse with tasteless artificial saliva (Figure 1C). In each run, low and high tastant concentrations were presented in a pseudorandomized order. Based on prior human psychophysical measurements (e.g., Low et al., 2017) and alert monkey stimulus-response electrophysiology recordings (Scott et al., 1994), the low and high concentrations were used to test whether reliably (high concentration) versus poorly (low concentration) detectable taste stimuli (and, hence, perhaps different degrees of “saliency,” “metabolic,” and/or “hedonistic” values) would impact the taste connectome differently. The low concentrations typically fail to produce behavioral correlates of detection or preference, and they are in the lowest range of concentrations triggering neuronal firing in NTS and the insula (Scott et al., 1986a,b).

A total of 276 runs (15 taste+rinse trial blocks per run) were collected from the eight individual macaque monkeys. These runs included 104 sour runs (37.68% of all runs), 90 salty runs (32.61%), and 82 sweet runs (29.71%) across all monkeys (Figure 1D, top panel). Note that all subjects received a balanced combination of taste qualities (*p* > 0.05, Chi-square test) with the exception of B09, C12, who received only sour and sweet, and G11 and B12 who received only one tastant − sour and salty, respectively (Figure 1D, bottom panel). This indicates a minimized influence of animal identity on the examination of taste quality differences.

Figure 1E illustrates the analytical steps employed to investigate the integrative nature of the taste connectome network. (1) The weight of each event type (or beta, β, coefficient) was extracted from the general linear model (GLM) regression of the BOLD signal time series for each run and taken to represent the relationship between the event and the BOLD signal within that region. (2) We then used the correlation between the β coefficients of region pairs as a proxy for their functional connectivity strength. (3) Then we examined inter-region connectivity and constructed an undirected graph between all connectome nodes using the functional connectivity matrix. (4) From the undirected graph we explored measures of centrality and modularity. The results obtained from these different steps are described in detail in the following sub-sections.

### Overall effects of taste concentration and quality on the β coefficient

**FIGURE 2:**
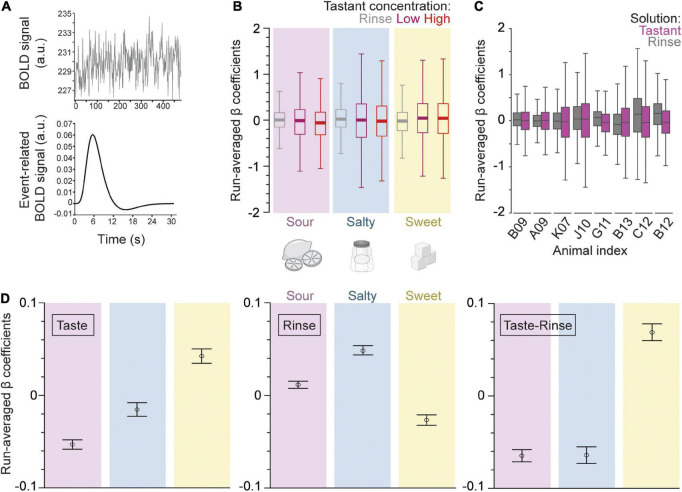
Gustatory connectome regions as a whole respond differentially to different taste qualities but not to taste concentrations. **(A)** The fMRI BOLD voxel signal time course was input into the beta-series general linear model (GLM). This time course is averaged across all voxels within a defined region space (upper panel, example from dAIC, or dorsal anterior insular cortex). The beta-series model outputs a convolved event-fitted BOLD response with amplitude equal to the beta weight for each trial presentation (lower panel). **(B)** Boxplot of the trial average β coefficients from the GLM model for low (magenta) and high (red) concentration tastant as well as rinse (gray) across sour (pink, *n* = 6,032), salty (blue, *n* = 5,220), and sweet (yellow, *n* = 4,756) taste runs, and across all regions taken together. Note the higher variance in the distribution of tastant as compared to rinse β coefficients across runs. The consistency of this effect is seen in the panel **(C)** boxplot of the trial average for rinse (gray) and tastant (magenta, low and high concentrations combined) β coefficients across individual macaque monkeys (*n* = 8 subjects; *n* = 290–3132 β coefficients per subject). Outliers were removed from the boxplots for visualization purposes in panels **(B,C)**. **(D)** Error bar plot of taste quality specific trial average β coefficients (mean ± SEM, *n* = 6032, 5212, 4756 for sour, salty, and sweet trials, respectively) for the taste (left panel) and rinse trial blocks (middle) as well as their difference (right). Note that sweet generates the largest response in the brain regions of the gustatory connectome (*p* = 5.1 × 10^– 37^, one-way ANOVA of taste and rinse difference).

Three event types (low tastant concentration, high tastant concentration, and rinse) were modeled as separate trial-wise parameter estimates for all runs analogously. Event-fitted β estimates were derived from the convolved BOLD signal time course (Figure 2A). The β-series was calculated for each of the three events separately, for each run, and for each of the 58 regions. The resulting averaged β-series represents the strength of the BOLD response (positive or negative) to each event in the context of the general linear model (GLM).

Figure 2B shows the boxplot distribution of the beta values averaged across all subjects and regions for each of the three events and each of the three tastants separately. For all taste qualities and tastant concentrations, the variance of the β values was about 2-fold greater for low and high taste concentrations as compared to rinse with artificial saliva (*p* < 10^–93^ for rinse vs. taste β variance comparison for all three taste qualities, two-sample *F*-test for equal variances, *n* = 16,000 trials). This indicates that the taste solutions had a stronger effect on the BOLD signal of the gustatory connectome regions than the rinse solutions, regardless of the taste quality and concentration. Thus, the IQR’s were 0.59 and 0.62 for run-averaged β coefficients of high and low concentration events of all tastes, respectively, and 0.35 for rinse.

The covarying BOLD activity for each taste quality at low and high concentrations was similar, with the exception of sour taste [Figure 2B; *t*-test for low vs. high concentration β coefficients of sour (*p* = 0.007), salty (*p* = 0.62), and sweet (*p* = 0.95)]. The effect of the sour concentration was, however, mild compared to the much stronger significant difference occurring between the taste and rinse β’s, regardless of the taste quality (Figure 2B; *p* < 10^–12^ *t*-test tastant vs. rinse β coefficients of all taste qualities). Finally, to further support the unique and similar effect of the taste stimuli compared to the rinse stimulus, we found a significant correlation between the average low vs. high concentration β coefficients within a run (Pearson’s *r* = 0.49; Supplementary Figure 1a). This correlation was absent between the average rinse and tastant β coefficients (Pearson’s *r* = 0; Supplementary Figure 1b).

Taken together, the results of this first analytical step demonstrate a rather robust similarity between the effects of low and high concentration tastant deliveries compared to artificial saliva. We used this overall similarity as a justification to combine the low and high concentration β coefficients in our subsequent analytical steps. Figure 2C shows the boxplot distribution of the beta values for each subject separately, and with the low and high taste concentrations pooled together, across all taste qualities and regions. The combined taste β distribution variance was again greater than rinse for all subjects (range of IQR change from 0.03 to 0.3), except one, C12 (−0.06 IQR change) (Figure 2C).

Next, we investigated the impact of taste quality on the β coefficients averaged within each run. Figure 2D shows the average betas (mean ± SEM) for each taste quality, as observed during the taste delivery event (left panel), during the rinse delivery event (middle panel), or when subtracting the betas of the rinse events from that of the taste events (right panel). During the taste delivery, we found the most positive average β from the sweet taste quality and lowest from the sour (Figure 2D, left panel; *p* = 2.6 × 10^–23^, one-way ANOVA, *n* = 16,000). We also quantified the β coefficients during the tasteless rinse solution delivery in between specific tastant delivery sessions. Surprisingly, we found significant differences with sweet generating the lowest coefficients, suggesting remaining effects from taste solutions (Figure 2D, center panel; *p* = 5.8 × 10^–26^, one-way ANOVA). Therefore, we decided to examine differences between taste qualities in reference to rinse as a measure of the tastant’s impact on the regions of the gustatory connectome. The change of BOLD-modeled β weights was most positive for sweet (0.068 ± 0.009 difference between rinse and tastant average β coefficients; mean ± SEM), and negative for both sour and salty (−0.065 ± 0.006 for sour, −0.064 ± 0.009 for salty; mean ± SEM). This indicates an overall slight, but significantly positive BOLD response for sweet and negative responses for sour and salty, as compared to artificial saliva (Figure 2D, right panel; *p* = 5.1 × 10^–37^, one-way ANOVA). Note that, while artificial saliva has been shown previously to act as a tasteless substance (Veldhuizen et al., 2007), the presentation of tastants prior to rinse may modulate post-taste responses (Bartoshuk et al., 1964).

### Effect of taste quality on the overall β-series correlation

**FIGURE 3:**
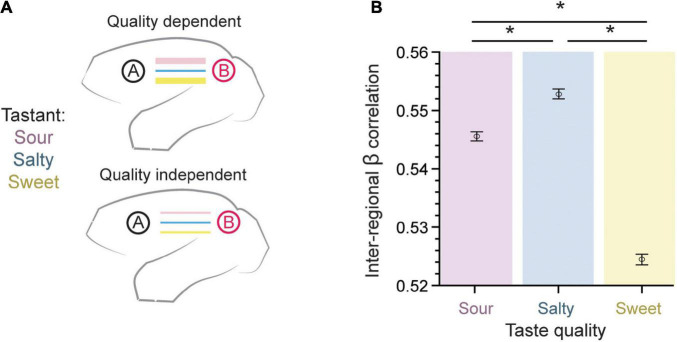
Inter-regional pairwise correlations across taste type. **(A)** The connection strength between pairs of brain regions measured using the β-series could either be dependent on the taste quality presented (top) or taste quality independent (bottom). **(B)** The error bar plot of inter-regional β coefficient correlation (mean ± SEM) is largest for salty and sour taste presentations as compared to sweet (*p* = 6.7 × 10^– 123^, one-way ANOVA, *n* = 456,228). This result supports a taste quality dependent model of connectivity within the gustatory connectome. *Indicates significant differences between pairs, with *p* < 10^−8^. Tukey’s post hoc test.

As a first approach to our beta-series correlation analysis, we sought to test whether taste quality modulates the strength of functional connection between brain regions as measured using the β-series (Figure 3A). For each region pair, the connection strength for each run was quantified using the Pearson’s correlation of their respective β coefficients (*n* = 15 per run). These correlations were then averaged using a Fisher’s z-transformation across runs (*n* = 276), so that an average correlation coefficient may be calculated (see *Methods: β-series correlation*). The inter-regional β coefficient correlation was largest for salty and sour taste presentations as compared to sweet (0.5456 ± 0.0008 for sour, 0.5528 ± 0.0008 for salty, 0.5244 ± 0.0009 for sweet, mean ± SEM; *p* = 6.7 × 10^–123^, one-way ANOVA, *n* = 456,228). This suggests a slight, but robust difference between taste qualities, with a lower overall inter-regional connectivity accompanying the positive BOLD response to sweet taste (compare Figures 2D, 3B).

### Significant callosal β-series correlations

**FIGURE 4:**
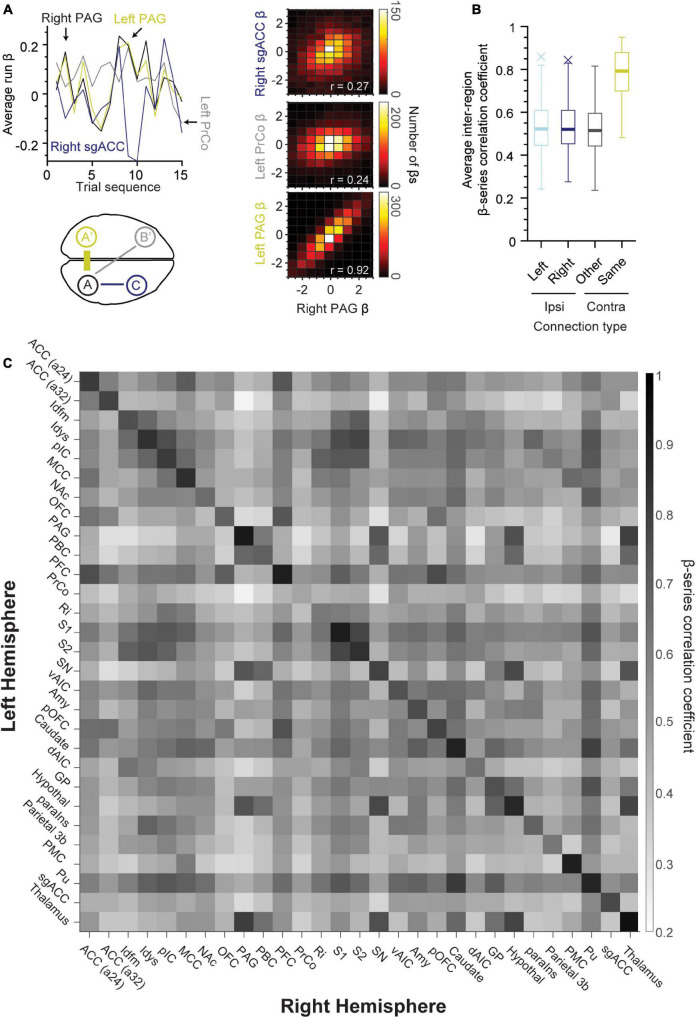
Same regions across hemispheres exhibit strong connectivity during taste processing. **(A)** Line plot of average run β-series demonstrating correlations between right PAG (upper left panel, A, black), left PAG (A’, yellow), left PrCo (B’, gray), and right sgACC (C, blue). Lower left panel is a schematic demonstrating the approximate location of these brain regions. Note that inter-hemispheric connections were strongest for the same brain region pairs (A and A’, or right and left PAG) as compared to other contralateral and ipsilateral connections. A 2D histogram (right panels) of the β coefficients for the same region pairs (lower panel, *r* = 0.92, left and right PAG, middle panel, *r* = 0.24, left PrCo and right PAG, upper panel, *r* = 0.27, right PAG and sgACC). **(B)** Boxplot of β correlation coefficients for ipsilateral (left hemisphere: light blue, *n* = 406; right hemisphere: dark blue, *n* = 406) and contralateral connections (same-name: yellow, *n* = 29; other regions: black, *n* = 812). **(C)** Heat map of the average β-series correlation coefficient between the region pairs in opposing hemispheres (*n* = 276 runs of sour, sweet and salty taste presentation). The existence of a darker main diagonal represents the stronger connection between each region and its contralateral counterpart as compared to other regions in the opposing hemisphere (97% of correlation coefficients on the main diagonal ≥ 0.60). ACC (a24, a32), area 24 and area 32 of the anterior cingulate cortex; Idfm, mid-dorsal fundus of the insular cortex; Idys, dysgranular insular cortex; pIC, posterior granular insular cortex; MCC, mid-cingulate cortex; NAc, nucleus accumbens; OFC, orbitofrontal cortex; PAG, periaqueductal gray; PBC, parabrachial complex; PFC, prefrontal cortex; PrCo, precentral opercular area; Ri, retro-insular cortex; S1, primary somatosensory cortex; S2, secondary somatosensory cortex; SN, substantia nigra; vAIC, ventral anterior insular cortex; Amy, amygdala; Caudate; dAIC, dorsal anterior insular cortex; GP, globus pallidus; Hypothal, hypothalamus; paraIns, parainsular cortex; Parietal (area) 3b; PMC, posterior medial cortex area 7m; Pu, putamen; sgACC, subgenual anterior cingulate cortex (area 25); Thalamus.

We examined the functional connectivity strength between regions of the left and right hemispheres. All region pairs exhibited correlations significantly different from zero even after Bonferroni-correction (*p* < 10^–19^, one-sample *t*-test). Homonymous (same left-right) region pairs exhibited the greatest correlation in comparison to non-homonymous contralateral region pairs as well as ipsilateral connections (schematized in Figure 4A, bottom left panel). The top left and right panels in Figure 4A show an example of this tendency, with the left PrCo and right sgACC (or subgenual anterior cingulate cortex) having weak correlations with the right PAG (Pearson’s *r* = 0.24 and 0.27, respectively, *n* = 4,140 β coefficients per region). Whereas, the right PAG exhibited strong correlations with its homonymous counterpart (right-left PAG, Pearson’s *r* = 0.92, *n* = 4,140 β coefficients per region). This difference in strength between homonymous “callosal” connections and other connections is further illustrated with the boxplot distributions in Figure 4B. The β coefficient correlation for the same region in the opposing hemisphere was significantly stronger (Figure 4B, yellow box, *n* = 29 region pairs, 97% of callosal correlations ≥ 0.60), as compared to other contralateral regions (Figure 4B, *n* = 1,653, *p* < 10^–52^, one-way ANOVA). Finally, Figure 4C shows a heat map matrix of correlation strength for all the left/right regions of the taste connectome, with a distinctly stronger correlation for the homonymous region pairs visible as a prominent main diagonal. Notably, the weakest callosal connection observed was with PrCo (Pearson’s *r* = 0.48). In general, across all tastants, the inter-hemispheric activity highlights a prominent role of PFC and the putamen (Pu) across connectome regions (Figure 4C). There were strong ipsilateral connections between S2 and Idfm (Pearson’s r: sour, 0.86; salty, 0.87; and sweet, 0.84) as well as between caudate and putamen (Pearson’s r: sour, 0.83; salty, 0.81; and sweet, 0.82).

### Modularity of the macaque gustatory connectome

**FIGURE 5:**
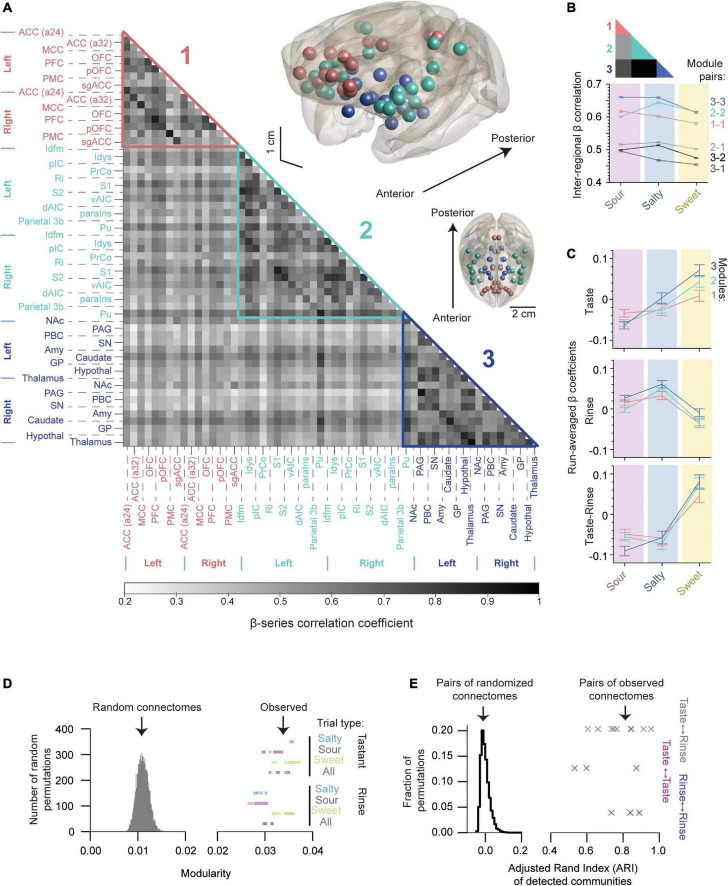
The gustatory connectome is partitioned into three interconnected sub-networks. **(A)** Grayscale heat map of average pairwise inter-regional β-series correlation in the taste connectome (*n* = 29 region pairs) with the detected modules highlighted. Three modules were revealed using the Louvain algorithm (modularity = 0.035): Module 1 (pink, *n* = 8 bilateral cortical regions), Module 2 (aqua, *n* = 12 bilateral cortical regions and putamen), and Module 3 (dark blue, *n* = 9 bilateral subcortical regions). Brain volume insets, shown in the upper right demonstrate the location of regions found within Modules 1 (pink spheres), 2 (aqua), and 3 (dark blue) relative to brain surface (gray transparent surface). **(B)** Line plot of average β-series correlation between modules (lower panel). Inset color codes the regions of matrix in panel **(A)** averaged to generate line plot. Region-wise correlations within and across modules indicate greater connectome-wide connectivity with sour and salty tastants as compared to sweet with the exception of Module 2-2 connections. **(C)** Line plot of β coefficients separated by detected modules (mean ± SEM, *n* = 4416, 6624, 4960 for Modules 1, 2, and 3, respectively). Note that the sweet taste generates the largest change in the β coefficients for all three modules. **(D)** Histogram of the modularity for random networks generated by permuting (*n* = 10,000) the weights of connections in panel **(A)**. Crosses indicate the observed modularity values of the gustatory connectome during tastant (upper, *n* = 44) and rinse (lower, *n* = 56) presentations separated by the type of taste quality. Each cross indicates a unique partitioning starting from different initial random ordering of the connectome regions (see also Supplementary Figure 2). Note that none of the random networks reach the modularity levels observed in the gustatory connectomes. **(E)** Histogram of Adjusted Rand Index (ARI) for 10,000 random networks indicating their partitioning similarity. Crosses indicate the observed ARI for modules detected under different taste quality (sour, salty and sweet) presentations for tastant (magenta, *n* = 3), rinse (blue, *n* = 3), and tastant-to-rinse (gray, *n* = 9). The vertical location of crosses has only a visualization purpose in panels **(D,E)**.

The gustatory connectome was assessed for the presence of communities (or modules) of interconnected regions using the Louvain community detection method (Blondel et al., 2008). Modularity is a measure of the relative density of links within as compared to between communities. Three communities were detected in the gustatory connectome of the macaque monkey. As illustrated in the grayscale correlation heat map in Figure 5A, Modules 1 (pink, *n* = 8 bilateral regions) and 3 (dark blue, *n* = 9 bilateral regions) contained only cortical and subcortical regions, respectively. Module 2 contained a combination of cortical regions and putamen (aqua, *n* = 12 bilateral regions). We detected these communities in the connectome for both taste and tasteless rinse trials (Supplementary Figure 2). Although the modules persisted during rinse, the level of modularity for gustatory connections during taste events was higher than the rinse (*p* = 4.3 × 10^–5^, two-sample *t*-test, *n* = 100 unique modularity values, crosses in Figure 5D).

The NMT v2 glass brain views in Figure 5A show the anatomical location of brain regions assigned to Modules 1 (pink spheres), 2 (aqua), and 3 (dark blue). Module 1’s cortical regions were located mainly medially along the cingulate gyrus and in the prefrontal cortex. Module 2’s cortical regions (and the putamen) were all located laterally, while Module 3’s regions were all subcortical. Of note, a preference was evident for connectivity between the insular parcellations and Module 2 as compared to Modules 1 and 3 (Supplementary Figure 3; *p* = 3.5 × 10^–45^, one-way ANOVA).

We measured the inter-modular average connectivity strength separated by taste quality and found stronger connections within modules as compared to between (Figure 5B, compare colored and gray scale lines). Comparing taste qualities, we found that region-wise correlations within and across modules indicate stronger connectivity with sour and salty tastants as compared to sweet. Interestingly, the sweet taste seemed to be consistently reducing the average level of connectivity at the full network and inter-modular levels with the only exception of Module 2-2 connectivity, which was weaker for sour than sweet (Figure 5B, *p* < 10^–16^, one-way ANOVA for all module pairs compared).

Pairwise relationships between gustatory connectome modules were examined for each of the taste qualities (Supplementary Table 1, upper table). Similarly, significance of beta weight differences between taste qualities (sour, salty, and sweet) were tested using one-way ANOVA followed by Tukey’s post hoc test in all three modules (1, 2, and 3; Supplementary Table 1, lower table). The ANOVA and pairwise multiple comparison *p*-values are tabulated for the tastants and rinse as well as for the tastant-rinse difference. Note that the one-way ANOVA revealed significant differences between taste qualities for each of the modules (Supplementary Table 1, lower table).

We quantified the β coefficients that indicate the strength of the BOLD response to the taste stimuli by the detected modules (Figure 5C, compare to Figure 2D) and found a similar pattern of the sweet taste generating the largest positive change in the β coefficients as compared to the rinse for all three modules. Grouping regions by the resulting three communities, we found that, overall, the network modules followed a similar pattern of activation, with positive responses to sweet and negative responses to the sour and salty taste presentations being present in all three modules (*p* < 10^–5^, one-way ANOVA for all three modules, *n* = 16,000 trials). We also found cases where different modules had differences in their beta coefficients, depending on the taste quality; however, these differences did not survive the subtraction of the rinse from the taste betas. For example, the subcortical module (Module 3) was most sensitive to the sweet taste presentation as compared to Modules 1 and 2 (Figure 5C, upper panel, one-way ANOVA, *p* = 0.0084), but this difference was nullified by the subtraction of the rinse betas (Figure 5C, bottom panel, one-way ANOVA, *p* = 0.26).

Since the order of brain regions considered by the community detection algorithm impacts the clustering, we repeated the detection of communities starting from different randomized sorting of regions (*n* = 10,000 random permutations, Supplementary Figure 2, left panel). After 10,000 permutations, we saw a labile (dynamic) positioning of the amygdala, caudate, nucleus accumbens, and sgACC most frequently within the sub-network assemblies. We found 99.87% of the detection iterations resulted in two possible community arrangements. Out of these two possible arrangements, we selected the community assignment with the highest level of modularity [modularity = 0.035, second most frequent (10.36%), last column in Supplementary Figure 2, left panel; see *Methods: Network modularity*].

We then sought to determine whether the modular structure in the connectivity graph could be observed in similar random networks. Therefore, we randomly permuted the connection weights of the connectivity matrix and found that none of the 10,000 permutations reached the level of modularity observed in the taste and rinse connectomes (Figure 5D, 0.011 ± 0.001, for random networks, mean ± SD, crosses denote the observed modularity for taste and rinse trials). This indicates that taste and rinse have distinct modular structures, stronger than any random permutation of the connections between nodes in the matrix.

Next, we wondered how consistent the clustering into sub-networks was for taste and rinse connectomes under the three presented taste qualities (sour, salty and sweet). For this, we measured the Adjusted Rand Index (ARI) of the communities detected for these connectomes (Figure 5E, crosses). To make sure this level of similarity between connectome communities could not be observed in similar, but random networks, we used 10,000 pairs of such networks and calculated the ARI of their community assignment. None of the random network pairs reached an ARI value as high as those obtained with the observed networks (histogram in Figure 5E, random network pair ARI of −0.0003 ± 0.024, mean ± SD).

**FIGURE 6:**
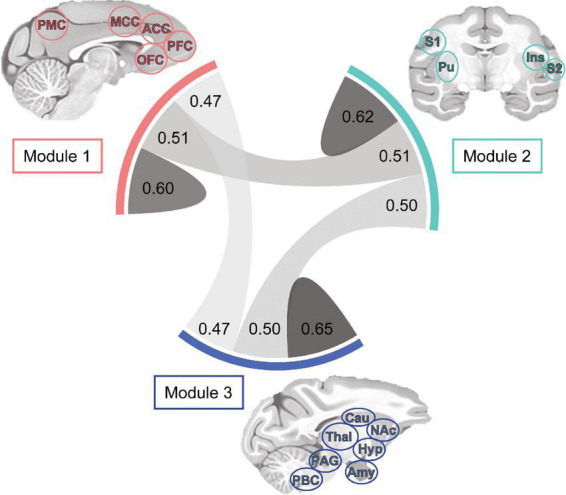
Tri-modular connectome and connection strength within and between sub-networks. Each module includes a unique set of regions and all modules exhibited bilaterality, indicating inclusion of region nodes in the left and right hemispheres. Chord diagram showing the correlation strength between bilateral regions of Modules 1 (*n* = 8), 2 (*n* = 12), and 3 (*n* = 9). Connection line thickness and darkness represent correlation strength. Note the difference in connection strength (*r*-value) between and within Modules: 1-1, 0.60; 1-2, 0.51; 1-3, 0.47; 2-2, 0.62; 2-3, 0.50; 3-3, 0.65. ACC, anterior cingulate cortex; Amy, amygdala; Cau, caudate; Hyp, hypothalamus; Ins, insula; NAc, nucleus accumbens; MCC, mid-cingulate cortex; OFC, orbitofrontal cortex; PAG, periaqueductal gray; PBC, parabrachial complex; PFC, prefrontal cortex; PMC, posterior medial cortex area 7m; Pu, putamen; S1, primary somatosensory cortex; S2, secondary somatosensory cortex; Thal, thalamus.

Finally, the density and strength of correlations across the macaque monkey gustatory connectome was assessed by plotting the chord diagram of modular connectivity (Figure 6, 0.60−0.65, 0.47−0.51 for intra-module vs inter-module connections, respectively). As expected, the intra-modular connectivity was stronger as compared to inter-module connections. However, this difference does not exclude still significant connections between the modules. This provides additional evidence for an interconnected gustatory connectome across three sub-networks.

### Centrality measures in the macaque taste connectome

**FIGURE 7:**
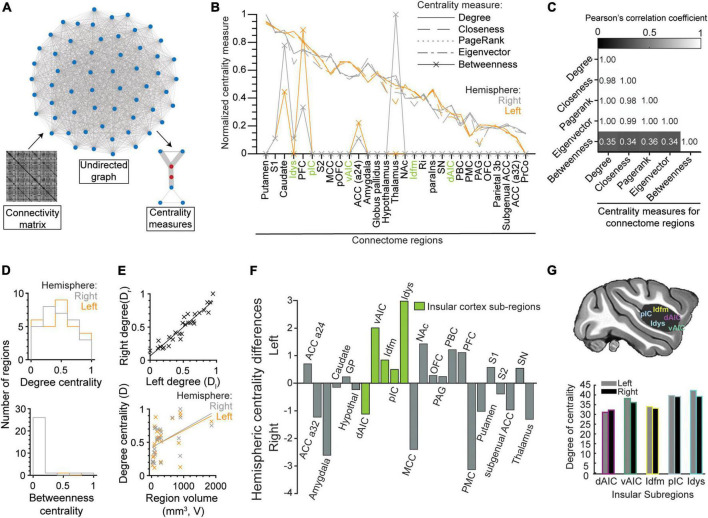
Centrality measures of brain regions in the gustatory connectome. **(A)** Schematics demonstrating brain region centrality measurement from an undirected graph constructed from the respective connectivity matrix of β-series correlation. **(B)** Line plot of the normalized (to the [0, 1] interval) degree (solid line), closeness (dashed line), PageRank (dotted line), eigenvector (dot-dash line), and betweenness (line with crosses) centrality measures for the connectome regions in the left (orange, *n* = 29) and right (gray, *n* = 29) hemispheres. Note that centrality measures correlate well with the degree centrality with the exception of betweenness. **(C)** Matrix of the Pearson’s correlation coefficient between five centrality measures. Numbers indicate the value of correlation. **(D)** Histogram of the distribution of the normalized degree centrality (upper panel) and betweenness centrality (lower panel) for the right (gray) and left (orange) connectome nodes. Note that the betweenness centrality is zero for most regions. **(E)** Scatter plots showing the correlation between right and left hemisphere degree centrality (upper panel, *r* = 0.95, linear fit line: D*_*r*_* = 0.87 × D_l_ + 0.06) and their relationship to the volume of the region (lower panel, left: *r* = 0.43, D_l_ = 2.5 × 10^– 4^ × V + 0.42, right: *r* = 0.49, D*_*r*_* = 2.8 × 10^– 4^ × V + 0.42). **(F)** Bar plot for difference between left and right degree centrality of sub-regions in the insular (green) and other connectome regions (gray). Note that degree centrality is skewed to the left hemisphere for insular cortical regions (*p* = 0.01, paired *t*-test of left vs. right degree centrality) and not the rest of the gustatory connectome (*p* = 0.55, paired *t*-test). All insular cortex sub-regions, except the dorsal anterior fundus (dAIC), had nodes with greater centrality in the left hemisphere. **(G)** Schematic of the brain showing the five regions-of-interest in the insular cortex (upper panel). Bar plot of the degree centrality for insular sub-regions with the dysgranular insular (Idys) cortex exhibiting the greatest degree centrality and the dorsal anterior insular cortex (dAIC) exhibiting the least (lower panel).

We used centrality measures to quantify the importance (i.e., centrality) of each node in the undirected functional connectivity graph derived from the beta-series correlation matrix (Figure 7A). The centrality measures included degree, closeness, PageRank, eigenvector, and betweenness (see Oldham et al., 2019 for a description of each measure). They were applied to each node/brain region separately. As illustrated in Figure 7B, the degree of normalized centrality was similar across all measures, except for betweenness. This observation was confirmed by the high correlations between degree, closeness, page-rank, and eigenvector (Figure 7C; all Pearson’s correlation coefficient > 0.98), and their low correlations with betweenness (Figure 7C; all Pearson’s correlation coefficient < 0.36).

Given the similarity between degree, closeness, PageRank, and eigenvector, we decided to take degree centrality (mean ± STD: 34.58 ± 4.67), which equates to the sum of all edge weights that a node has within the connectome, hence reflecting the number of highly significant correlations (or functional connections) that each node possesses. As shown in Figure 7B, the striatum (putamen and caudate), primary somatosensory area (S1), including area 3a, dysgranular insular cortex (Idys) and prefrontal cortex (PFC) were the regions with five highest degrees of centrality, in both hemispheres (see also Supplementary Table 2), which was to be expected based on the higher proportion of high correlations that these regions have with other brain regions, within and outside of their modules (Figure 5A).

Unlike degree centrality, betweenness centrality identifies nodes with the highest number of times that they occupy the shortest path between two regions. Since our graph is densely connected with the shortest paths usually represented by the direct edge connecting two nodes, only 10 connectome nodes had a non-zero betweenness centrality (Figure 7B; see also Figure 7D, lower panel). These nodes included the right and left caudate (right > left) (but not the putamen), PFC (left > right), area 24 of the ACC (a24), and the right thalamus, as well as left and right S1, and right Idys to a lesser extent. The high density of short paths inherently limits the relevancy of the betweenness measure; it was nevertheless reported here for the sake of completeness.

To further characterize the degree centrality, we closely analyzed differences between left and right nodes. At first sight, there was a significant correlation between the bilateral region pair centralities (Figure 7E, upper panel, Pearson’s *r* = 0.95); and, when considering the entire connectome, we were unable to find a significant difference between centrality measures of the left and right counterparts of a region (Figure 7D upper panel, *p* = 0.55, paired *t*-test, *n* = 29 region pairs and *n* = 5 centrality measures). However, when considering only the insular sub-regions, we found an asymmetric centrality, with all insular regions having a greater centrality on the left side, except for dAIC [Figure 7F, *p* = 0.01, paired *t*-test, *n* = 5 insular sub-regions (green), *n* = 5 centrality measures]. Among the insular regions, the dysgranular insular region exhibited the greatest degree centrality (Idys; left: 41.86, right: 38.88), with the dorsal anterior sub-region exhibiting the least (Figures 7F,G; degree centrality left: 30.90, right 32.02). The select arrangement of hemispheric dominance, by centrality measures, indicates a potential predominant role for the left insular cortex in taste processing (see *Discussion: Leftward laterality in the insular centrality*).

Finally, we tested whether the volume of the regions could account for higher centrality. We obtained a positive correlation between volume and degree centrality (Figure 7E, lower panel, *r* = 0.43, 0.49 for left and right regions, respectively). This suggests a more central role for larger brain regions, although numerous small (e.g., Idys) and medium (e.g., caudate) regions still reached high degrees of centrality, comparable to the largest two regions (i.e., PFC and S1).

## Discussion

Within the growing field of connectomics, there is an evident gap in available studies on chemosensory networks (Veldhuizen et al., 2022). A contemporary review of published chemosensory connectivity research in humans identified only 15 studies to have examined gustatory networks by fMRI (Farruggia et al., 2022). It has been noted, however, that several properties of human brain connectivity are conserved across a wide range of species (Assaf et al., 2020), a finding further substantiating research in other mammals (Ohla et al., 2019). Here, the study of fundamental principles of connectomics is aided through the use of macaque monkeys, a species capable of providing a substantive comparative basis for how the primate brain processes gustatory information.

We evaluated the connectome for gustatory processing in the anesthetized macaque monkey using correlation analysis of taste-evoked beta (β) coefficients and graph theory measures of modularity, centrality, and laterality. We identified three modules (medial forebrain Module 1, lateral forebrain Module 2, and subcortical Module 3) with far greater modularity strength than any randomly assigned clusters, which remained stable across taste qualities and concentrations. Although the modules persisted during rinse, the level of modularity for gustatory connections during taste events was significantly higher than the rinse.

Taste qualities had a slight, but robust effect on the overall direction of the taste-evoked response, and the mean strength of region-to-region connectivity was differently modulated by sweet (positive responses, weaker correlations) versus sour and salty (negative responses, stronger correlations). Centrality measures identified several hubs, particularly the striatum, dysgranular insula, prefrontal and somatosensory cortices, which may relate to ongoing sensory information processing. Compared to the dysgranular insula hub, the anterior insula and cingulate nodes, while being interconnected, had only a moderate role in inter-module connectivity. Laterality measures confirmed the classically strong “callosal” connection of same-name regions (Margulies et al., 2007; Wu et al., 2022). They also suggested a leftward dominance in taste connectivity in the insula.

### Three consistent “taste” connectome modules

The three modules observed in the present study remained relatively stable across taste qualities and also occurred in response to rinse. This robustness, and the simple fact that all the regions included in the gustatory connectome also process a vast array of other sensory modalities and executive functions, means that our tri-modular “taste” connectome is not specific to taste, but serves a much broader purpose, likely in the context of salience. Two observations indicate nonetheless that the modules can be regulated by the nature of the sensory stimulus. First, while modules formed during both taste and rinse, the beta coefficients of the low and high tastant concentrations were highly correlated with one another, but not with the beta coefficients of rinse. This is remarkable because, while there was no effect of the concentration per se (except for a slight effect with the sour taste), the gustatory connectome still responded more strongly to the low taste concentration than to rinse, even if the low taste concentration was enough to trigger neuronal responses in the insula and NTS (Scott et al., 1986a,b), but not high enough to allow accurate taste detection and recognition in humans (Low et al., 2017). This suggests that the three modules occur across all events, but may be reinforced in the presence of even slightly more salient events. Second, the fact that taste qualities differently affected the beta coefficients and inter-regional connectivity (i.e., beta coefficient correlations) indicates that distinct sensory modalities and sub-modalities could lead to activity and connectivity fluctuations within the connectome, which could be tested by employing a broader variety of stimuli (e.g., Li et al., 2022).

A recent graph theory analysis based on resting-state fMRI using seed regions responding to noxious heat (47.5°C) in lightly sedated macaques identified six “heat pain” sub-networks, including at least one medial cortical module (ACC and PFC), three lateral cortical modules, including insular and somatosensory cortices, and one subcortical module including thalamus and caudate (Wu et al., 2022), that all together bear some resemblance to our three taste modules. Like in our study, bilateral anterior insular cortex (AIC) regions were strongly interconnected, but fail to act as hubs. Unlike in our study, their ACC acted as a hub with strong ties to the posterior insula and S2. A study in humans, using a similar stimulus, identified four modules: lateral sensorimotor (including insula, S1, S2, but also the medial cingulate cortex, MCC), medial frontoparietal (ACC, PCC, and PMC), lateral frontoparietal, and subcortical limbic (amygdala and hippocampus) (Fauchon et al., 2020). The lateral sensorimotor, medial and, to some extent, subcortical modules closely resemble our lateral forebrain Module 2 (baring the inclusion of the human MCC), medial forebrain Module 1, and subcortical Module 3, respectively. In this awake human study, the painful stimulus further enhanced the role of AIC as a major connector hub. Differences in the exact definition of the modules and strength of correlations, particularly for AIC and ACC, likely reflect differences in a priori parcellation or region selection, the nature of the stimulus (i.e., innocuous taste vs. noxious heat; see also Li et al., 2022), and the level of “alertness,” from deep opioid anesthesia (present study) to light ketamine sedation (Wu et al., 2022), to full alertness (Fauchon et al., 2020). Nevertheless, the distinction of three spatially distinct functional regions (lateral forebrain, medial forebrain, and subcortical) appears to be robust and deserves further consideration.

Several of the regions selected to build the gustatory connectome belong to common functional networks. For instance, AIC and ACC are core hubs of the salience network (Seeley, 2019), which overlaps with the central autonomic network (Benarroch, 1993), the cingulo-opercular network (Dosenbach et al., 2007), and the interoceptive-allostatic system (Kleckner et al., 2017). These “limbic” networks involve also top-down and bottom-up interactions with autonomic and interoceptive subcortical centers. Most of the regions of the medial forebrain module, including ACC, PCC, and PMC, belong to the default mode network (Vincent et al., 2007). The somatosensory cortices form the lateral sensorimotor network to which the insula, in particular the posterior insula, has also been associated (Cauda et al., 2011a; Shen et al., 2019). The tendency to recognize medial and lateral components within (i.e., salience) and between (e.g., default-mode vs. sensorimotor) these networks is reminiscent of the neuroanatomical definition of medial and orbitolateral prefrontal networks in macaque monkeys (Price, 2007; Price and Drevets, 2012). The medial network is made of architectonic areas that are mono-synaptically connected to numerous higher-order poly-modal and non-sensory cortical areas, including various prefrontal areas and the whole rostro-caudal extent of the medial cingulate gyrus down to the retrosplenial cortex, which all together comprise our medial Module 1, as well as various regions of the temporal lobe that were not considered here. In contrast with the medial network, the orbitolateral network connects with a constellation of sensory areas, including all that were assigned here to the lateral Module 2. At the functional level, this dichotomy could reflect a balance of cooperation and/or competition (Cocchi et al., 2013) between medial task maintenance and lateral sensory processing triggering task/network switching through the activation of the AIC-ACC hub connection of the salience network (e.g., Johnston et al., 2007; Sridharan et al., 2008; Menon and Uddin, 2010). Both the medial and orbital prefrontal networks project densely to subcortical autonomic centers (Freedman et al., 2000; Price, 2007), suggesting that both Modules 1 and 2 can work in parallel to regulate sensory gating and efferent control in Module 3.

### Network hubs

In graph theory, brain regions with high centrality are interpreted to act as hubs that favor and potentially control communication between many nodes, within and/or between modules (Sporns et al., 2007; Oldham and Fornito, 2019). In the anesthetized macaque receiving taste stimuli, the most central regions likely to act as hubs were the striatum (putamen and caudate), somatosensory cortex, and dysgranular insula, all bilaterally. Unlike the dysgranular insula, vAIC, and ACC had centrality values above average that remained, however, moderate; dAIC ranked in the lower end.

The striatum has been associated with various functional networks, including as a hub in the cingulo-opercular network (Dosenbach et al., 2007; Tu et al., 2012). The striatum receives dense, inter-digitated and overlapping projections from numerous cortical regions (Selemon and Goldman-Rakic, 1985), including the ACC, PFC, insula and somatosensory cortex, making it a substrate for the integration of critical poly-modal information (Chikama et al., 1997; Tang et al., 2020). This integrative role appears crucial, since the “hubness” of the striatum decreases proportionally with the increase in the magnitude of schizophrenic symptoms, for example (Tu et al., 2012). The strong connectivity of the putamen with most of the regions of the gustatory connectome suggests that it maintains this integrative role under anesthesia. While not flagged as highly central (caudate being an exception), the nucleus accumbens was, along with the amygdala, caudate and sgACC, a rather labile region that could easily switch between Modules 2 and 3, supporting its role as a functional pivot integrating motivation and reward in the context of goal-directed behavior (Cauda et al., 2011b).

The primary somatosensory cortex (S1) has an uncertain role in taste. Its area 3b receives mechanoreceptive afferents from the tongue and oral cavity, and its fundal area 3a receives minor projections from the posterior and basal subdivisions of the ventromedial thalamus, VMpo and VMb, respectively (Craig, 2002; Kaas, 2005). S1 is a broad region, and it was not further parcellated to isolate a lateral region of area 3a most likely representing the tongue. Irrespective of its putative role in taste representation, the involvement of S1 most likely relates to its role as a central hub conveying mechanoreceptive and proprioceptive information within the broader somatosensory network (Gundlach et al., 2020). This involvement may, however, change when comparing distinct sensory modalities (interoceptive vs exteroceptive) and perhaps with additional controls (e.g., an additional “rinse” block that would not be affected by the prior taste solution delivery).

### Insular sub-regions

In the present study, architectonic areas of the insula (Evrard et al., 2014) were grouped into five regions reflecting prior functional parcellations of the human insula (e.g., Kurth et al., 2010; Kelly et al., 2012; Nomi et al., 2018). Each of these regions process various functional modalities (Simmons et al., 2013), and taste processing involves more than just one of these regions (Small, 2010; Yeung et al., 2017; Ohla et al., 2019). While all insular regions were connected within the lateral forebrain Module 2, the dysgranular insula (Idys) had the highest degree of centrality, along with the striatum and S1. This supports its role in integrating taste with poly-modal afferents from many cortical and subcortical regions (Mesulam and Mufson, 1982; Evrard and Craig, 2015; Evrard, 2019). Next to Idys, the posterior insula region (pIC) had the highest insular centrality. The pIC region contained Idfp, Igd, and Igv (see Supplementary Figure 4). While Idfp, the cortical terminus of the lateral spinothalamic tract via VMpo, connects with somatosensory areas (Evrard, 2019), it may, like Idfm, have too specialized connections to act as a hub. The high centrality of pIC was most likely due to the poly-modal (interoceptive, somatosensory, and auditory) nature of Igd and Igv (Evrard, 2019).

Both vAIC and dAIC form critical hubs with a crucial role in task and network switching (Sridharan et al., 2008; Menon and Uddin, 2010), and preferential roles in affective and cognitive representations, respectively (Kurth et al., 2010; Evrard, 2019). Although they were assigned to different modules, ACC and vAIC had a similar degree of centrality, slightly above average, and were moderately connected to one another. Together, with the presence of von Economo neurons in both vAIC and ACC (Evrard et al., 2012; Evrard, 2018), this supports the idea that, similar to humans, macaque monkeys do have a ventral salience network (Touroutoglou et al., 2016), with vAIC and ACC likely orchestrating communication between medial and lateral networks during salience-driven task switching (Menon and Uddin, 2010). While repeated presentation of taste exposes variability in the neural response magnitude (Di Lorenzo and Victor, 2003), the use of a repeated sensory stimulus (Poellinger et al., 2001; Wagner et al., 2006) and anesthesia likely dampened the salient nature of the stimulus and, hence, the correlation of vAIC and ACC. In addition, the taste event design may have engaged the more sensory-driven Idys (Wu et al., 2021), dynamically relocating connectome hubs.

In contrast to vAIC, dAIC did not figure among the highly correlated nodes. This supports prior evidence that monkeys lack a dorsal salience network (Touroutoglou et al., 2016), perhaps due to their disproportionately smaller size compared to the human dAIC (Bauernfeind et al., 2013). An increasing amount of evidence implicates the human dAIC in the interoceptive shaping of cognition and in the homeostatic gating of access of brain activity to cognitive control and consciousness (Nelson et al., 2010; Warnaby et al., 2016; Wu et al., 2019; Molnar-Szakacs and Uddin, 2022). This implication was recently further evidenced by the correlation between the anesthetic loss of behavioral response and loss of dAIC response to stimuli in humans (Warnaby et al., 2016; Huang et al., 2021). Despite its small size, the macaque dAIC harbors signs of cognitive activity (Wang et al., 2020) and could be a smaller primal homolog of the human dAIC (Evrard, 2018, 2022). However, in the present study, any such implication would have most likely been blocked by the anesthesia (Warnaby et al., 2016; Giacometti et al., 2021; Huang et al., 2021), and, as for vAIC, by the dampening of salience by the repetitious nature of the stimulus.

### Leftward laterality in the insular centrality

Within the connectome, bilateral homonymous (i.e., same name) region pairs exhibited the strongest functional connectivity, which is consistent with numerous prior reports (e.g., Margulies et al., 2007; Wu et al., 2022). The three modules did not show any marked asymmetry, however, centrality measures showed that most insular regions had a slight but robust leftward dominance, which is reminiscent of prior studies suggesting a leftward representation of taste in the dorsal insular cortex of humans (Yeung et al., 2017). This asymmetric representation did not appear clearly in a more recent report of multiple taste clusters in the human insula, although there was one right dorsal anterior insular activation that did not occur in the left hemisphere (Avery et al., 2020). In the present study, the dorsal anterior insula was the only sub-region to have a rightward centrality, which could relate to a dominant role of the right AIC (Craig, 2005); although making such association in the present experimental context is merely anecdotal and warrants proper investigation.

### Functional connectivity under anesthesia

We found a highly correlated connectome for taste in the macaque monkey; the absence of anti-correlations is reminiscent of previously reported resting-state network properties under anesthesia (Uhrig et al., 2016). Anesthetized fMRI signals exhibit a more static functional connectivity matrix – one resembling the underlying structural connectivity closer than an active, conscious state network (Barttfelda et al., 2015; Tasserie et al., 2022). Comparing structural connectivity in the adult human brain with the anesthetized macaque gustatory connectome, we see that both have identified the bilateral putamen as a network hub (Oldham and Fornito, 2019). It would be of interest to see if the putamen plays an equally central role in the awake macaque monkey gustatory connectome.

As already indicated above, the use of an opioid anesthetic affects the level of autonomic arousal and thalamocortical activity as well as top-down (Laureys et al., 2009; Aru et al., 2019) and bottom-up (Luo et al., 2018) input on the characterization of sensory afferent information. It also weakens functional connectivity, including in the cingulo-opercular connections (Giacometti et al., 2021), and can alter the organization of the functional networks (Moeller et al., 2009). Although not documented in detail, the effect of the anesthesia was particularly clear in one subject (C12) with prolonged anesthesia, and with reduced beta coefficient values, closer to those of the rinse events. Thus, caution must be applied when interpreting the results. Nevertheless, prior reports showed that anesthesia, including with opioids, did not block the correlation between ripple events and BOLD signal (Logothetis et al., 2012), the expression of the default mode (Vincent et al., 2007) and fronto-intraparietal (Moeller et al., 2009) networks, electrophysiological and BOLD signal response to innocuous and noxious cutaneous stimuli, which consistently produce activity in the dorsal fundus of the insula, as well as in the anterior insula, at least for the most salient stimuli (e.g., noxious pinch of the skin) (Evrard et al., 2009; Craig, 2010). As evidenced by the differences in beta coefficients derived from sour, salty and sweet taste qualities as well as rinse in the present study, it is unlikely that the opioid anesthesia completely blocked the cortical processing of taste afferents. Nevertheless, based on human and monkey studies, it is obvious that anesthesia was one of the two possible factors that contributed to the low beta coefficient in dAIC and low connectivity in dAIC and, to a lesser extent, vAIC. As highlighted above, another factor might have been the use of repeated stimuli, which will reduce novelty and salience.

## Conclusion

The basis of chemosensory processing depends on interactions between brain regions across a distributed network. The properties of the macaque monkey gustatory connectome uncovered with this work purport evidence for a highly interconnected, positively correlated network comprised of three sub-networks operating densely within their community partitions. Taste processing occurs in regions (e.g., insula) that process several other modalities and activities. This enables us to conclude that the three bilateral modules observed here likely serve more than taste processing. Yet, the marked effect of taste over rinse delivery indicates that taste plays a significant role in the activity fluctuations observed in the gustatory connectome. The present study provides the first connectivity and graph theory examination of taste processing in a non-human primate. While performed under anesthesia, this examination appears to provide valid clues on the dynamic functional neuroanatomy of gustatory processing. These clues are relevant, not only to the fields of chemosensory and metabolic neuroscience, but also to the examination of interoception via shared pathways, with great implications for emotional and cognitive processing.

## Materials and methods

### Animal welfare and ethical approval

The present study was conducted with 8 adult rhesus macaque monkeys [*Macaca mulatta*; 4 females; average weight (mean ± STD): 9.04 ± 2.04 kg]. Animals were handled according to the guidelines of the European Parliament and Council Directive 2010/63/EU on the protection of animals used for scientific purposes. The local animal welfare and ethics authorities (*Regierungspraesidium*) reviewed and approved the ethical protocol underlying this research study.

### Anesthesia

All macaques fasted for ∼12 h prior to anesthesia for neuroimaging experiments. The anesthesia procedure employed here was previously described in detail (Logothetis et al., 2012). Efforts were made to monitor stress responses as well as suitable parameters for imaging changes in the BOLD signal. To attain physiological values optimal for fMRI, body temperature was maintained at 38.3−38.8°C using a rectal thermometer and a water-heating pad. End tidal CO_2_ and oxygen saturation were also kept constant at 33 mmHg and >95%, respectively. Anesthesia was maintained using a combination of remifentanil (1−3 μg/kg/min) and mivacurium chloride (5−7 mg/kg/h). The physiological state of the animal was continuously monitored using infrared pulse oximetry (Nonin Medical Inc., Plymouth, MN, United States), electrocardiography (ECG), thermometry, and sphygmomanometry. Animals were granted a minimum of 2 weeks between anesthetized experiments to fully recover.

### Taste stimulus preparation and delivery apparatus

Tastants were prepared using household products at room temperature. The taste qualities used and their low/high molar concentrations were as follows: sour (citric acid, C_6_H_8_O_7_), 0.00125/0.08 M; salty (sodium chloride, NaCl), 0.025/0.8 M; and sweet (sucrose, C_12_H_22_O_11_), 0.015/0.5 M. A tasteless solution that mimics the concentration of saliva was used as the base for all taste solutions and as the rinse solution (12.5% dilution of artificial saliva; stock: 25 mM KCl, 2.5 mM NaHCO_3_ in dH_2_0; Veldhuizen et al., 2007).

To deliver tastants we utilized a custom-built Peripheral Suction Fluid Stimulator (PSFS). The PSFS was comprised of a small basin supporting the underbelly of the tongue and a funnel at the external portion of the basin, which facilitated the removal of infused liquid by vacuum suction. Liquid infusions entered the mouth via an infusion line embedded within the mouthpiece used to help fix the jaw and head during scanning. The liquid drop trajectory targeted the anterior third of the tongue.

### Functional magnetic resonance imaging design paradigm

Anesthetized fMRI data were acquired across 18 days of imaging using a 7T vertical bore MRI scanner (Bruker, Billerica, MA, United States) equipped with a 60 cm (inner diameter) imaging gradient system. Once positioned in a custom-designed MR-compatible chair, anesthetized animals were connected to physiological monitoring sensors; their legs were wrapped from toe to pelvis to prevent venostasis; the torso and arms were wrapped snugly in towels to maintain temperature and prevent obstruction of blood flow and airways. The head of the animal was immobilized using fitted ear bars and mouthpiece (the same mouthpiece supporting the PSFS delivery system). In between taste runs, BOLD signal and anesthesia levels were intermittently checked both by the physiological readouts and by the examination of single fMRI scans using a visual flicker stimulus as a subcortical signal localizer (Logothetis et al., 1999). The collection of taste data was completed within a larger experimental framework on chemosensory and interoceptive processing; in effect, on the same day either before or after taste blocks animals participated in additional non-gustatory runs not relevant for the current study on the taste connectome and thus not further described.

Liquid solution infusions were delivered via syringe pumps (Aladdin-1000, World Precision Instruments), programmed to operate at the same flow rate (17 mL/min). The stimulation paradigm began with a 7 sec taste infusion period (resulting in 1.98 mL of taste solution being delivered), followed by a 2 sec delay period to capture residual drips from the delivery spout, then a final 7 sec of rinse infusion (i.e., 1.98 mL of artificial saliva), and finally a 15 sec inter-trial pause, before restarting the same sequence (Figure 1C). Each functional scan sequence was 8 min in total with 15 trials of pseudo-randomized presentations of low and high taste concentrations. In a given experiment day, a target of 8 runs per taste quality were sought (120 trials), whereby same-quality runs were recorded in sequential imaging blocks and the presentation order of different taste qualities was randomized. The number of runs acquired overall was contingent upon the subject’s physiological stability as well as time allocated to acquire BOLD scan data either before or after the taste runs (in a pre-specified, randomized order).

### Image acquisition

Whole-brain volumes were acquired either by multi-shot (2 segments) gradient-echo EPI (1 × 1 mm in-plane nominal resolution; TR/TE: 2000/19 ms; 20 axial slices; acquisition matrix: 96 × 96; reconstruction matrix: 128 × 128) in 7 animals or by a signal-to-noise efficiency optimized parallel gradient-echo EPI (0.75 × 0.85 mm in-plane nominal resolution; TR/TE: 1000/18 ms, 18 axial slices; acquisition and reconstruction matrix: 128 × 113; acceleration factor in the phase encoding dimension: 2; GRAPPA-reconstruction of the missing spatial frequencies) in 3 subjects using 8-channel phased-array receivers exclusively manufactured and rigidly mounted on each individual head-fixation helmet. The following parameters were consistent across runs: flip angle: 53°; FOV: 96 × 96 mm; slice thickness 2 mm; ∼150 kHz data sampling frequency. Slice volumes were acquired in contiguous sections. During each experiment, a T2*-weighted anatomical scan encoding the same volume as the functional runs was collected to image the native structural space (FOV: 96 × 96 mm; matrix size: 256 × 256; 8 segments; flip angle: 8°; TR/TE: 4000−6500/25−48 ms; 40 slices).

### Image preprocessing and normalization

Using SPM12 (Statistical Parametric Mapping; Wellcome Department of Imaging Neuroscience, London, United Kingdom), each functional scan was realigned to its first volume with six rigid-body transformation parameters, and then registered to its corresponding native structural scan. The structural scans were normalized to a symmetric population-template, NMT v2 (Jung et al., 2021), using a combination of DARTEL diffeomorphic warping (Ashburner, 2007) and an in-house routine using a linear local-weighted mean approach to fit the positioning of anatomical structures between the template and single-subject images (MATLAB 2017b, MathWorks, Natick, MA, United States). The resulting transformation matrix was then applied to the functional scans (already spatially registered to the subject’s anatomical scan). At each step the spatial alignment was manually examined by visual inspection. An analysis of the resulting spatial disparity between functional images and template was below the size of the smoothing kernel applied (FWHM = 2 mm).

### Selection of brain regions for the gustatory connectome

The regions (*n* = 29) selected for the connectome modeling (Figure 1A and Supplementary Table 2) were determined prior to analysis and based upon human (Faurion et al., 1998; O’Doherty et al., 2001; Small et al., 2003; Kringelbach et al., 2004; Ogawa et al., 2005; Rolls, 2009; Spetter et al., 2010; Veldhuizen et al., 2011) and non-human primate literature (Plata-Salamán and Scott, 1992; Scott and Plata-Salamán, 1999; Kadohisa et al., 2004; Verhagen et al., 2004). This provides an empirical foundation for studying the involvement of each region in the macaque taste connectome. The regions, as listed in Supplementary Table 2, encompass the insular cortex sub-regions, orbitofrontal, anterior and cingulate cortices, and amygdala (Kadohisa et al., 2004, 2005a,b). Five insular sub-regions were considered here in an attempt to obtain a finer differentiation of insular connectivity in the gustatory connectome. The macaque insular cortex was previously subdivided into 15 distinct architectonic areas, including seven agranular areas (Iam, Iai, Ial, Iap, Iapm, Iapl, and Ivfa), four dysgranular areas (Idd, Idm, Idv, and Ivfp), and four granular areas (Idfa, Idfp, Igd, and Igv) (Evrard et al., 2014). An additional granular area, Idfm (or Idfa-c), was recently inserted in between Idfa (or Idfa-r) and Idfp (Evrard, 2019). This new area Idfm is most likely the primary cortical recipient of taste afferents relayed by the basal part of the ventromedial nucleus of the thalamus (VMb) (Pritchard et al., 1986; Hartig et al., 2017); whereas, Idfa putatively receives mainly visceral afferents and only a small fraction of gustatory afferents (Evrard, 2022). In the present study, selected insular areas were grouped into five regions: ventral anterior insular region (vAIC: Iam + Iai + Ial + Iapm), dorsal anterior insular region (dAIC: Iapl + Idfa), middle dorsal fundus (Idfm), posterior insular region (pIC: Idfp + Igd + Igv), and dysgranular insular region (Idys: Idd + Idm + Idv + Ivfp) (Supplementary Figure 4).

Considering both ascending and descending projections along the taste processing hierarchy, the gustatory connectome coalesces into a collection of subcortical and cortical regions as nodes of probable involvement. Nodes include structures of the basal ganglia (i.e., substantia nigra, globus pallidus), striatum (i.e., caudate, putamen, nucleus accumbens), hypothalamus and secondary somatosensory cortex. The posterior medial cortex area 7m, inclusive of the precuneus region, was also considered (Margulies et al., 2016). Primary somatosensory cortex (S1) was included since it provides tactile sensory input to polymodal regions, such as the dysgranular insula, and its known recipient region area 3a of gustatory thalamocortical relay projections (Craig, 2015; Evrard, 2018). Even though thalamic relay of gustatory afferents is localized to the basal subdivision of the ventromedial (VMb) thalamic nucleus (Pritchard et al., 1986), the entire thalamic volume was added as a node (with the exception of the geniculate nuclei) to include afferent and efferent processing sub-divisions. Lastly, the nucleus tractus solitarius (NTS, first central relay of taste information in the medulla oblongata, Beckstead et al., 1980; Yaxley et al., 1985) was not included in this connectome since it was outside the imaging field-of-view. Retro-insula (Ri), posterior OFC (area 13), and parietal area 3b were included for exploration given prior anatomical work suggesting inter-connections between these regions and insular as well as frontal cortices (Neal et al., 1987; Öngür and Price, 2000; Uyanikgil et al., 2018). The other two regions (paraIns and precentral opercula area; PrCo) were included since they were neighboring connectome regions and bore relevance, given the 2 mm^3^ voxel size, on the BOLD response of their neighboring voxels.

The gustatory connectome was generated by extracting the desired regions-of-interest (ROIs) from the CHARM (Jung et al., 2021) and SARM (Hartig et al., 2021), which are respectively, cortical and subcortical digitized atlases of the rhesus macaque. The regions were extracted from the atlas volumes and coalesced, along with the manually parcellated ROIs of the insular cortex sub-divisions, into an aggregate gustatory (taste) connectome mask using the fslmaths image calculator (Analysis Group, FMRIB, Oxford, United Kingdom; Smith et al., 2004). A manual parcellation of five insular cortex sub-divisions was done using MRICro as the original insular cortex parcellation in the CHARM was based upon a coarse parcellation scheme implemented by (Saleem and Logothetis, 2007). The parcellated sub-divisions are the granular posterior (pIC), dysgranular (Idys), granular mid- (Idfm) and anterior dorsal (dAIC), and agranular ventral anterior (vAIC) insular cortex.

### β-series derivation

We used data acquired from delivering solutions containing 3 different taste qualities: sour, salty and sweet (Figure 1C). A general linear model (GLM) was fit to the voxel-wise blood-oxygen-level-dependent (BOLD) signal measured by fMRI to calculate the weight of the solution delivery event [β coefficients, model of the form: Y = Xβ+ε, Y: BOLD signal, X: design matrix, ε: residual error (Figure 1E)]. The first-level design matrix contained, for each run separately, two covariates-of-interest (low and high concentration taste stimulation) and one regressor for control (rinse using artificial saliva). These regressors were the result of convolution of a boxcar function (width = 7 sec, equivalent to infusion duration) with the canonical hemodynamic response function (HRF, peaking 6 sec after stimulus onset).

Nuisance regressors were included in the GLM design matrix to model confounding variables. For each trial sequence, a 2 sec residual period between taste offset and rinse onset was included as the baseline (B_0_) nuisance regressor as well as the six demeaned rigid-body motion correction transformation parameters estimated during preprocessing. The BOLD signal time series was detrended by applying a 128 sec high-pass filter.

Each run contained 15 trials of low and high concentration tastant presented in a pseudorandomized order. The GLM model was fit to each taste trial independently creating 15 individual tastant and rinse β weights for each run (β-series). This provided a way to measure sensitivity to inter-trial variations. Subject-wise first-level analysis of the β-series models was performed in SPM12.

To compute the average run β (Figure 2), we calculated separately the average β coefficients for taste (*n* = 15 per run with low and high concentration tastes presented in a pseudorandomized manner) and rinse solution trials (*n* = 15 per run). We had 276 runs with 58 regions within each run that resulted in 16,000 (after excluding the left and right amygdala from 4 runs in monkey B09 due to low signal-to-noise) averaged taste and rinse solution βs. The distribution of the average run β separated by taste quality and animal identity was displayed as boxplots in Figures 2B,C. The mean and standard error of the mean separated by taste quality and detected modules was calculated to create the line plots in Figures 2D, 5C. We also visualized the relationship between low and high taste concentration and rinse β coefficients by plotting their 2-dimensional histograms as a heat map (Supplementary Figure 1). To compare variances of the taste and rinse β coefficients, we used a two-sample *F*-test for equal variances (Figure 2B).

### β-series correlation

The functional connectivity of 29 bilateral regions included in the gustatory connectome was examined across both hemispheres of the brain. For all voxels within each region, the regression coefficients (β weights) as computed by the GLM model, were averaged.

The β-series correlation processing was based on the beta-series correlation (BASCO) toolbox (Göttlich et al., 2015) and previously published methods for ROI-based functional connectivity analysis in humans (Rissman et al., 2004; Ranganath et al., 2005) and rodents (Winkelmeier et al., 2022). In the context of experimental designs with many stimulus repetitions, such as in the current study, it has been suggested that the β-series method is more statistically powerful as compared to the psychophysiological interaction (PPI) method (Cisler et al., 2014).

We used the Pearson’s correlation coefficient of the β-weights for each pair of regions (*n* = 58 regions, *n* = 1,653 unique region pairs) to measure their connectivity strength (*n* = 15 weights per run, *n* = 276 runs). To average correlation coefficients across runs, we applied Fisher’s z-transformation (inverse hyperbolic tangent function) to obtain normally distributed and variance stabilized values (Fisher, 1915). The averaged correlation coefficients were transformed back to the [−1, 1] range using the hyperbolic tangent function. The standard error of the mean (SEM) was also measured with the Fisher z-transformed correlation coefficients. The error bar extents were then transformed to the original [−1, 1] range for visualization (Figures 3B, 5B).

We mostly used the average β-series correlation from taste delivery trials for community detection (Figures 5A,B, 6 and Supplementary Figures 2, left panel, 3, shuffled distributions in Figures 5D,E). However, we also detected modules for connectivity matrices separated by taste qualities as well as for when the tasteless rinse solution was delivered (Supplementary Figure 2, right panel, crosses in Figures 5D,E).

The connectivity strength of insular sub-regions (Idfm, Idys, pIC, vAIC, and dAIC) to regions from all three sub-modules was plotted as a boxplot in Supplementary Figure 3. The significance of connection strength difference was measured using a one-way ANOVA, followed by Tukey’s honest significant test (*p* = 3.5 × 10^–45^).

The order in which the nodes are considered by the community detection algorithm affects the final output. Therefore, we used 10,000 random permutations of nodes as starting points to calculate the robustness of the communities. The results of the community detection on the randomly shuffled ordering of nodes for connectivity graphs of taste and rinse trials is displayed in Supplementary Figure 2, along with their respective modularity values.

To compare the modularity of the observed gustatory connectome to random networks, we randomly shuffled the weights on the average connectome of the taste trials (graph in Figure 5A). We then measured the modularity of the clustering from community detection in the random networks and plotted the histogram in Figure 5D (*n* = 10,000 permutations).

We also tried to understand the level of similarity between clustering arising from connectivity measured from different taste qualities. To obtain a measure of clustering similarity we used the Adjusted Rand Index (ARI), which quantifies the similarity in clustering with identical partitions resulting in a value of 1. We also used 10,000 random network pair partitions to get the distribution of random ARI values (see histogram in Figure 5E).

### Network laterality

We compared the strength of callosal Fisher-transformed correlation coefficients between a region and its contralateral counterpart versus other regions in the ipsi- and contra-lateral hemispheres (Figure 4B) using a one-way ANOVA (*p* = 8.55 × 10^–53^). We also plotted the correlation coefficients for all of the inter-hemispheric connections as a matrix (Figure 4C). All callosal region pairs had correlation coefficients that were significantly different from zero (one-sample Student’s *t*-test against zero, Bonferroni-corrected for 1,653 comparisons). The average β coefficients for three region pairs (right PAG with left PAG, left PrCo, and right sgACC) were displayed as a line plot to demonstrate the difference between low and high levels of correlation (Figure 4A, left panel). A 2D histogram of all the β coefficients for the same three region pairs was also visualized as a heat map (Figure 4A, right panel).

### Network modularity

In order to determine if modules exist within the graph of the taste connectome, the Louvain community detection algorithm was applied. The aim was to find groups of nodes that were more internally connected than externally. Modularity measures the density of links inside communities compared to links between communities, a scalar value between -0.5 (non-modular clustering) and 1 (fully modular clustering). In the Louvain community detection, small communities are formed by optimizing modularity through grouping of nodes starting from a randomized ordering. Then, the small communities are merged in a hierarchical manner to increase modularity. The process is stopped when no further increase is possible. This community detection method optimizes modularity as the algorithm progresses and it is competitive with similar heuristic algorithms in terms of processing speed (Blondel et al., 2008). Detected communities were visualized by chord diagrams plotted using the *circlize* package in R (Gu et al., 2014).

### Network centrality

The average connectivity matrix of the 29 bilateral regions calculated from the taste delivery trials was converted to a weighted undirected connectivity graph. The degree, closeness, PageRank, eigenvector and betweenness centrality graph measures (Oldham et al., 2019) were then applied to determine central nodes in the taste connectome of the macaque monkey as previously described (Lohmann et al., 2010; van den Heuvel and Sporns, 2011; Harriger et al., 2012; Zuo et al., 2012). Since degree centrality correlated well with the other centrality measures in the gustatory connectome, except for betweenness centrality (Figures 7C,D), we examined the effects of region volume and hemisphere differences using degree centrality (Figures 7E-G). Degree centrality is a simple and understandable measure that is calculated by summing the edge weights connected to each node.

To test for significance of the difference between the left and right hemisphere centrality measures, we used a paired Student’s *t*-test on the five centrality measures of the five insular sub-regions (*p* = 0.01, *n* = 25 for both hemispheres*).* The same analysis was applied to all the brain regions of the gustatory connectome as well (*p* = 0.55, *n* = 145 for both hemispheres*)*. All code for analysis was written in MATLAB 2021a (MathWorks, United States) and Python 3.9.

## Data availability statement

The original contributions presented in this study are included in the article/Supplementary material. All analysis software and data used for figure generation are publicly available at: https://gitlab.mpcdf.mpg.de/alik/Brain_network/. The connectivity matrices are available as CSV files here: https://gitlab.mpcdf.mpg.de/alik/Brain_network/ -/tree/master/python/data/connectivity_matrices. Further inquiries can be directed to the corresponding authors.

## Ethics statement

The present study was conducted with eight adult rhesus macaque monkeys (*Macaca mulatta*; four females; average weight (mean ± STD): 9.04 ± 2.04 kg). Animals were handled according to the guidelines of the European Parliament and Council Directive 2010/63/EU on the protection of animals used for scientific purposes. The local animal welfare and ethics authorities (*Regierungspraesidium*) reviewed and approved the ethical protocol underlying this research study.

## Author contributions

RH and HCE conceived the experimental design. RH performed experiments. RH and AK designed and implemented analyses, validation, and visualization. All authors contributed to writing the manuscript.

## Supplementary material

Supplementary Figure 1 Correlation between tastant beta coefficients is absent between rinse and taste. **(a)** A 2D histogram of the joint distribution of the mean low and high concentration taste event β coefficients demonstrating their interdependence (Pearson’s *r* = 0.49) separated by taste quality (sour: pink, salty: blue, sweet: yellow). Color intensity indicates the number of runs with the indicated average low and high concentration β coefficients. **(b)** Same as **(a)** for the average rinse and taste events revealing no significant correlation (Pearson’s *r* = 0).

Supplementary Figure 2 The most frequently identified assembly of networks within the taste connectome is tri-modular. The robustness of modularity was tested by detecting communities starting from random initial ordering of brain regions and measuring the fraction of identical partitions. Matrix of module identities for connectome regions during taste solution presentation **(left)** and tasteless rinse **(right)**. Note the similar robustly tri-modular organization.

Supplementary Figure 3 Interaction between insular sub-regions and gustatory connectome modules. Boxplot of the connection strength (correlation coefficient of β-series) between insular sub-divisions (*n* = 10, Idfm, Idys, pIC, vAIC, and dAIC in both hemispheres) to regions in the three modules of the taste connectome (*n* = 160, 230, 180 correlation coefficients for Modules 1, 2, and 3, respectively).

Supplementary Figure 4 Anatomical insular cortex parcellation scheme. The colored contours illustrate the regions of the insular cortex used for the beta-series correlation analysis in the present study. Each of these regions contained one or several architectonic areas previously defined by cyto- and myelo-architectonic examination of the macaque monkey insula (Evrard et al., 2014). The selected insular areas were grouped into five regions: ventral anterior insular region (vAIC: Iam + Iai + Ial + Iapm), dorsal anterior insular region (dAIC: Iapl + Idfa), middle dorsal fundus (Idfm), posterior insular region (pIC: Idfp + Igd + Igv), and dysgranular insular region (Idys: Idd + Idm + Idv + Ivfp). IPS, inferior peri-insular sulcus; L, limen; POC, primary olfactory cortex; SPS, superior peri-insular sulcus.

Supplementary Table 1 Gustatory connectome sub-network comparisons. Pairwise relationships between gustatory connectome modules were examined for each of the taste qualities **(upper table)**. Similarly, significance of beta weight differences between taste qualities (sour, salty, and sweet) were tested using a one-way ANOVA followed by Tukey’s post hoc test in all three modules (1, 2, and 3, lower table). The ANOVA and pairwise multiple comparison *p*-values are tabulated for the tastants and rinse as well as for the tastant-rinse difference. Note that the one-way ANOVA revealed significant differences between taste qualities for each of the modules **(lower table)**.

Supplementary Table 2 Gustatory connectome region coordinates, volume, and degree centrality. The taste connectome is comprised of 29 bilateral regions. The center-of-mass coordinates for each region is provided along with the degree centrality measurement. Data for both left and right hemisphere measurements are denoted as left/right (L/R) values. The left and right regions are symmetric. The anatomical regions are listed in alphabetical order and their relevant acronym is shown in parentheses.
