# Supplementary figures and images for: Interconnected sub-networks of the macaque monkey gustatory connectome
Source: Front Neurosci. 2023 Feb 16;16:818800. doi: 10.3389/fnins.2022.818800 (PMC9978403; doi:10.3389/fnins.2022.818800)

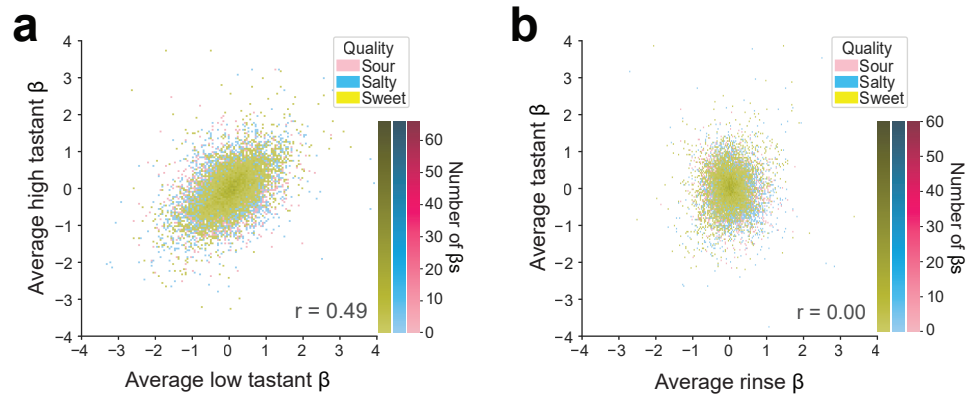

Suppl. Figure 1  
Hartig et al. 2022

Supplement: Supplementary Figure 1 — Correlation between tastant beta coefficients is absent between rinse and taste. (a) A 2D histogram of the joint distribution of the mean low and high concentration taste event β coefficients demonstrating their interdependence (Pearson’s r = 0.49) separated by taste quality (sour: pink, salty: blue, sweet: yellow). Color intensity indicates the number of runs with the indicated average low and high concentration β coefficients. (b) Same as (a) for the average rinse and taste events revealing no significant correlation (Pearson’s r = 0). [file Image_1.PDF]

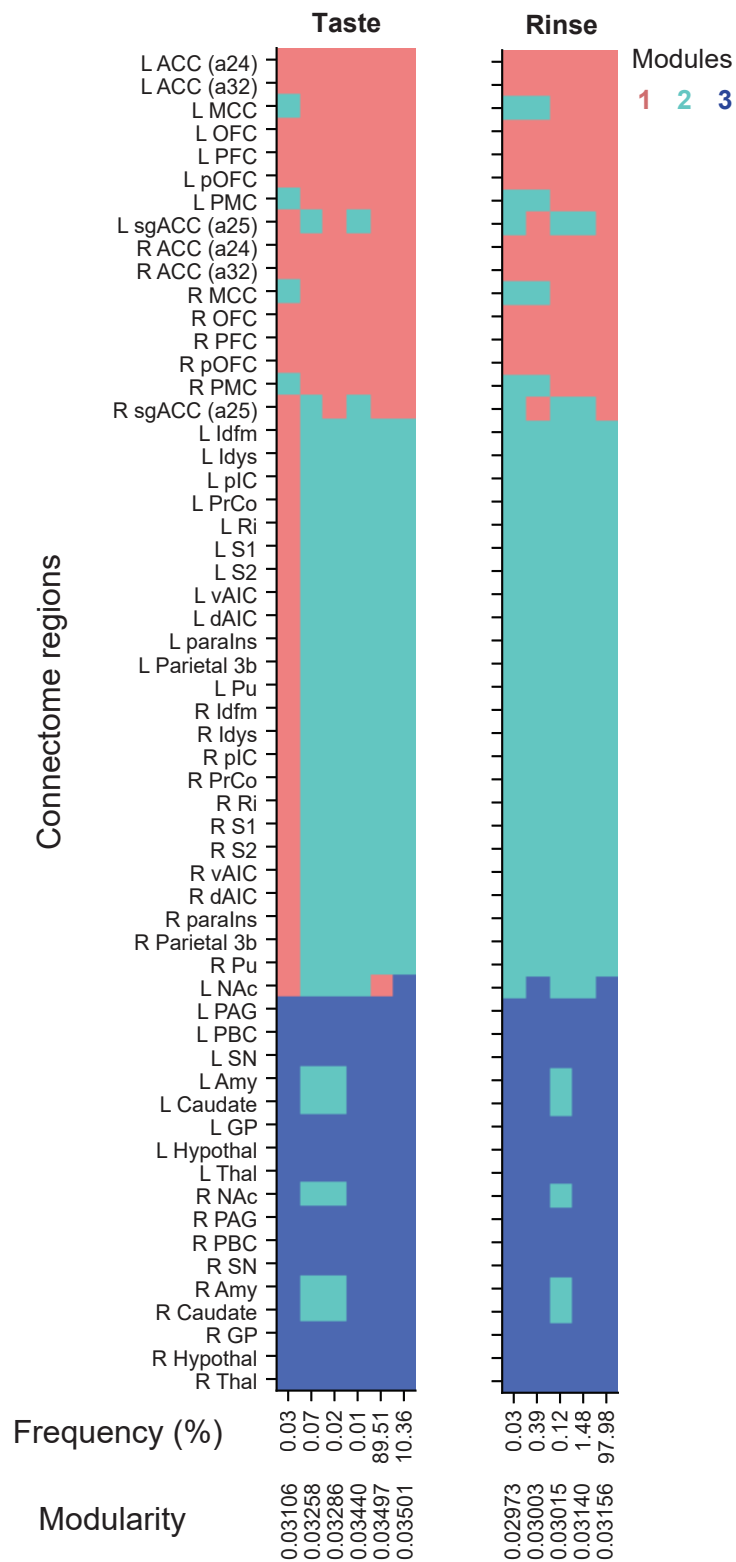

Randomly seeded partitions  
(n = 10,000 random seeds)

Suppl. Figure 2  
Hartig et al. 2022

Supplement: Supplementary Figure 2 — The most frequently identified assembly of networks within the taste connectome is tri-modular. The robustness of modularity was tested by detecting communities starting from random initial ordering of brain regions and measuring the fraction of identical partitions. Matrix of module identities for connectome regions during taste solution presentation (left) and tasteless rinse (right). Note the similar robustly tri-modular organization. [file Image_2.pdf]

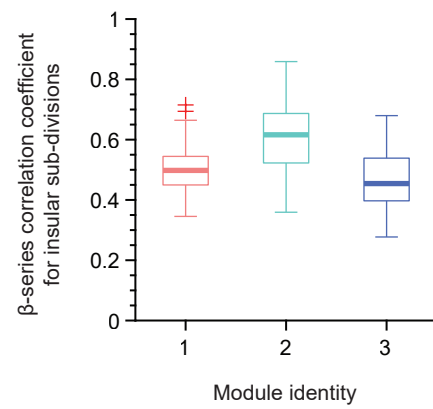

Suppl. Figure 3  
Hartig et al. 2022

Supplement: Supplementary Figure 3 — Interaction between insular sub-regions and gustatory connectome modules. Boxplot of the connection strength (correlation coefficient of β-series) between insular sub-divisions (n = 10, Idfm, Idys, pIC, vAIC, and dAIC in both hemispheres) to regions in the three modules of the taste connectome (n = 160, 230, 180 correlation coefficients for Modules 1, 2, and 3, respectively). [file Image_3.pdf]

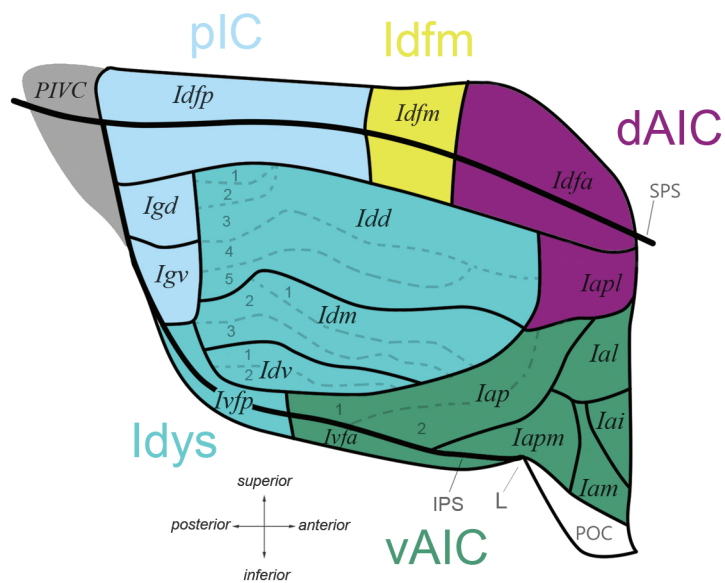

Suppl. Figure 4  
Hartig et al. 2022

Supplement: Supplementary Figure 4 — Anatomical insular cortex parcellation scheme. The colored contours illustrate the regions of the insular cortex used for the beta-series correlation analysis in the present study. Each of these regions contained one or several architectonic areas previously defined by cyto- and myelo-architectonic examination of the macaque monkey insula (Evrard et al., 2014). The selected insular areas were grouped into five regions: ventral anterior insular region (vAIC: Iam + Iai + Ial + Iapm), dorsal anterior insular region (dAIC: Iapl + Idfa), middle dorsal fundus (Idfm), posterior insular region (pIC: Idfp + Igd + Igv), and dysgranular insular region (Idys: Idd + Idm + Idv + Ivfp). IPS, inferior peri-insular sulcus; L, limen; POC, primary olfactory cortex; SPS, superior peri-insular sulcus. [file Image_4.pdf]
