# Supplementary material for: Interconnected sub-networks of the macaque monkey gustatory connectome
Source: Front Neurosci. 2023 Feb 16;16:818800. doi: 10.3389/fnins.2022.818800 (PMC9978403; doi:10.3389/fnins.2022.818800)
Supplement: Supplementary Table 1 — Gustatory connectome sub-network comparisons. Pairwise relationships between gustatory connectome modules were examined for each of the taste qualities (upper table). Similarly, significance of beta weight differences between taste qualities (sour, salty, and sweet) were tested using a one-way ANOVA followed by Tukey’s post hoc test in all three modules (1, 2, and 3, lower table). The ANOVA and pairwise multiple comparison p-values are tabulated for the tastants and rinse as well as for the tastant-rinse difference. Note that the one-way ANOVA revealed significant differences between taste qualities for each of the modules (lower table). [file Table_1.pdf]

		Tastant		Rinse		Tastant-Rinse	
**Taste quality**	Modules compared	ANOVA *p*-value	Pairwise multiple comparison *p*-value	ANOVA *p*-value	Pairwise multiple comparison *p*-value	ANOVA *p*-value	Pairwise multiple comparison *p*-value
**Sour**	Module 1, Module 2	0.05927833	0.13836134	0.00646228	0.16311209	0.03115659	0.92016683
	Module 1, Module 3		0.06290750		0.49832730		0.04658019
	Module 2, Module 3		0.86502576		0.00507861		0.06929311
**Salty**	Module 1, Module 2	0.22931486	0.97167613	0.09460562	0.30607241	0.72496687	0.80131170
	Module 1, Module 3		0.27471851		0.08014664		0.99868099
	Module 2, Module 3		0.31303929		0.67186415		0.75796150
**Sweet**	Module 1, Module 2	0.00844952	0.17607873	0.12646530	0.96418870	0.26474352	0.36417096
	Module 1, Module 3		0.00565880		0.16570115		0.28514520
	Module 2, Module 3		0.26177927		0.19391565		0.96271200

		Tastant		Rinse		Tastant-Rinse	
**Modules**	Tastes compared	ANOVA *p*-value	Pairwise multiple comparison *p*-value	ANOVA *p*-value	Pairwise multiple comparison *p*-value	ANOVA *p*-value	Pairwise multiple comparison *p*-value
**Module 1**	Sour, Salty	0.03270833	0.88183783	$2.95868x10^{-07}$	0.32935699	$1.73093x10^{-06}$	0.89488223
	Sour, Sweet		0.03232506		0.00012846		$3.51948x10^{-05}$
	Salty, Sweet		0.11764191		$3.53381x10^{-07}$		$9.38466x10^{-06}$
**Module 2**	Sour, Salty	$2.74241x10^{-10}$	0.04120260	$1.13343x10^{-13}$	$9.61541x10^{-07}$	$8.17177x10^{-17}$	0.60152071
	Sour, Sweet		$1.07945x10^{-10}$		0.01137922		$1.46508x10^{-12}$
	Salty, Sweet		0.00011282		$7.56687x10^{-14}$		$7.01413x10^{-15}$
**Module 3**	Sour, Salty	$1.23477x10^{-15}$	$7.16825x10^{-05}$	$1.521x10^{-07}$	0.01109567	$7.56933x10^{-17}$	0.22731044
	Sour, Sweet		$1.9464x10^{-16}$		0.00768209		$1.61798x10^{-16}$
	Salty, Sweet		0.00016151		$5.98116x10^{-08}$		$2.57745x10^{-10}$
