# Supplementary material for: Interconnected sub-networks of the macaque monkey gustatory connectome
Source: Front Neurosci. 2023 Feb 16;16:818800. doi: 10.3389/fnins.2022.818800 (PMC9978403; doi:10.3389/fnins.2022.818800)
Supplement: Supplementary Table 2 — Gustatory connectome region coordinates, volume, and degree centrality. The taste connectome is comprised of 29 bilateral regions. The center-of-mass coordinates for each region is provided along with the degree centrality measurement. Data for both left and right hemisphere measurements are denoted as left/right (L/R) values. The left and right regions are symmetric. The anatomical regions are listed in alphabetical order and their relevant acronym is shown in parentheses. [file Table_2.pdf]

Gustatory Connectome Region Coordinates, Volume and Degree Centrality

Anatomical Region	Hem.	Center-of-mass x	y	z	Unilateral Volume (mm^3)	Degree Centrality
Anterior Cingulate Cortex (ACC)						
Area 24	L/R	3.08	31.3	27.1	289.89	36.75/36.05
Area 32	L/R	1.47	34.6	21.6	91.61	27.73/28.96
Amygdala	L/R	9.68	18.9	6.58	302	35.06/37.67
Caudate	L/R	6.19	20.3	20.6	649.56	41.56/41.71
Globus Pallidus	L/R	8.96	16.5	14.6	233.39	36.24/36.01
Hypothalamus	L/R	2.94	15.3	11.3	178.08	35.35/35.58
Insular Cortex						
dAIC	L/R	18.7	24.3	16.4	77.84	30.90/32.02
vAIC	L/R	13.1	26.2	13.3	169.45	37.84/35.83
Idfm	L/R	19.1	19.7	18.6	91.41	33.76/32.92
pIC	L/R	17.2	11.8	18.8	96.34	39.29/38.79
Idys	L/R	18.9	18.2	14.3	129.45	41.86/38.88
Mid-Cingulate Cortex (MCC)	L/R	2.98	16.5	28.4	188.38	36.09/38.49
Nucleus Accumbens (NAc)	L/R	4.6	24.1	13.7	94.97	34.38/32.95
Orbitofrontal Cortex (OFC)	L/R	11.1	36.3	20.7	288.97	29.36/29.08
ParaIns	L/R	18	19.6	10.6	27.28	34.04/31.44
Parietal Lobule Area 3b	L/R	21.9	3.28	28.4	97.59	29.09/28.86
Posterior OFC (area 13)	L/R	7.2	32.5	18	419.17	37.69/36.38
Periaqueductal Gray (PAG)	L/R	1.21	3.77	11.5	49.48	29.36/29.12
Parabrachial Complex (PBC)	L/R	3.94	0.99	5.68	45.84	30.74/29.52
Prefrontal Cortex (PFC)	L/R	9.34	34	26.4	1865.19	40.62/39.51
PrCo	L/R	18.3	27.6	13.8	95.95	25.54/28.22
Posterior Medial Cortex (area 7m)	L/R	1.72	-7.02	27.2	894.06	27.90/31.03
Putamen	L/R	12.2	18.7	16.4	895.11	43.10/44.12
Primary Somatosensory Cortex (S1)	L/R	14.8	7.09	29.5	900.69	42.33/41.76
Retro-Insula (Ri)	L/R	14.2	6.7	21.9	15.11	32.65/33.31
Secondary Somatosensory Cortex (S2)	L/R	21	15	19.4	269.67	38.06/38.45
Subgenual ACC (area 25)	L/R	1.42	27.8	16.2	32.89	28.39/29.36
Substantia Nigra (SN)	L/R	4.59	9.85	8.6	111.22	31.91/31.37
Thalamus	L/R	5.66	9.61	17	848.28	34.65/35.96
